# Iron oxide nanoparticle-mediated hyperthermia stimulates dispersal in bacterial biofilms and enhances antibiotic efficacy

**DOI:** 10.1038/srep18385

**Published:** 2015-12-18

**Authors:** Thuy-Khanh Nguyen, Hien T. T. Duong, Ramona Selvanayagam, Cyrille Boyer, Nicolas Barraud

**Affiliations:** 1 Centre for Advanced Macromolecular Design (CAMD) and Australian Centre for NanoMedicine (ACN), School of Chemical Engineering, The University of New South Wales, Sydney 2052, Australia; 2 Centre for Marine Bio-Innovation, School of Biotechnology and Biomolecular Sciences, The University of New South Wales, Sydney 2052, Australia

## Abstract

The dispersal phase that completes the biofilm lifecycle is of particular interest for its potential to remove recalcitrant, antimicrobial tolerant biofilm infections. Here we found that temperature is a cue for biofilm dispersal and a rise by 5 °C or more can induce the detachment of *Pseudomonas aeruginosa* biofilms. Temperature upshifts were found to decrease biofilm biomass and increase the number of viable freely suspended cells. The dispersal response appeared to involve the secondary messenger cyclic di-GMP, which is central to a genetic network governing motile to sessile transitions in bacteria. Furthermore, we used poly((oligo(ethylene glycol) methyl ether acrylate)-*block*-poly(monoacryloxy ethyl phosphate)-stabilized iron oxide nanoparticles (POEGA-*b*-PMAEP@IONPs) to induce local hyperthermia in established biofilms upon exposure to a magnetic field. POEGA-*b*-PMAEP@IONPs were non-toxic to bacteria and when heated induced the detachment of biofilm cells. Finally, combined treatments of POEGA-*b*-PMAEP@IONPs and the antibiotic gentamicin reduced by 2-log the number of colony-forming units in both biofilm and planktonic phases after 20 min, which represent a 3.2- and 4.1-fold increase in the efficacy against planktonic and biofilm cells, respectively, compared to gentamicin alone. The use of iron oxide nanoparticles to disperse biofilms may find broad applications across a range of clinical and industrial settings.

Bacteria have an extraordinary ability for adaptation, activating defence systems when put under stress, while thriving when the conditions improve. This capacity is often the cause of failed antibiotic and antimicrobial therapies with dramatic consequences both in healthcare and industrial contexts^1^. The study of bacterial adaptive behaviours and the molecular mechanisms that regulate them is crucial to develop novel effective antimicrobial strategies to treat infectious diseases and control contaminations.

In nature bacteria predominantly live in biofilms, which are sessile multicellular communities comprised of bacterial cells encased in a matrix of secreted polymeric substances. The biofilm mode of growth confers bacteria with increased resistance and persistence to external stress. If a biofilm successfully forms on host tissues, it often leads to chronic infections that antimicrobial treatments, which have been traditionally designed against planktonic bacteria, as well as immune defences fail to fully eradicate^2^. Biofilms have been linked to 80% of infections, forming on living tissues (e.g. respiratory, gastrointestinal and urinary tracts, oral cavity, eyes, ears, wounds, heart, cervix) or indwelling medical devices (e.g. dialysis catheters, prosthetic implants, contact lenses)^3^. In the industry, formation of biofilms can cause economic loss, for instance by inducing fouling and corrosion of wet surfaces or clogging of filtration membranes^4^. It can also pose a threat to public health by acting as reservoirs of pathogens in drinking water distribution systems^5^.

The biofilm lifestyle appears to be the main phase of a biphasic lifecycle, of which the other phase is the single-celled, dispersive planktonic lifestyle. Bacteria have the ability to switch between these two states via transition stages of attachment or dispersal. While the attachment stage has been well documented in several species, the reverse transition of dispersal has been much less studied despite its interest for developing novel strategies to control and remove unwanted biofilms^6^. Dispersal, which consists in the coordinated release of single cells or small aggregates from the interior of mature biofilms, is a key adaptive strategy that allows bacterial communities to spread and colonize new surfaces^7^. Recent studies have shown that biofilm dispersal can be induced in response to a range of environmental cues including changes in the availability of nutrients such as carbon sources^8^,^9^ or oxygen (O_2_)^10^, low levels of nitric oxide (NO)^11^ or iron levels^12^, as well as several bacterially derived signals including quorum sensing (QS) acyl-homoserine lactones^13^, autoinducing peptides^14^ and diffusible fatty acids^15^,^16^. Furthermore, the regulation of biofilm dispersal involves intracellular secondary messengers such as nucleotides cyclic di-GMP (c-di-GMP) and cyclic AMP^7^. c-di-GMP is the central element of a complex regulatory network that also includes sensor and effector proteins and controls the transition between planktonic and biofilm lifestyles^17^. Via this network, environmental cues and signals can stimulate enzymes that modify intracellular levels of c-di-GMP, which in turn regulates a range of downstream effectors leading to biofilm formation or dispersal. Diguanylate cyclases (DGC) that synthesize c-di-GMP typically increase biofilm formation when high intracellular levels of c-di-GMP induce the production of extracellular matrix polysaccharides or fimbriae adhesins. Conversely, phosphodiesterases (PDE) that degrade c-di-GMP favour a motile planktonic lifestyle by inducing the degradation of the biofilm matrix components and expression of flagella and chemotaxis genes^17^. Thus biofilm dispersal appears to be an active, highly regulated process responsive to changes in environmental conditions.

The role of temperature variation, a common cue for life cycle transitions in eukaryotic ecology^18^, has received little attention in biofilm studies so far. Several reports suggest that it may be involved in regulating biofilm to planktonic transitions. Early work showed that oral bacteria such as *Neisseria subflava, Haemophilus* and *Streptococcus* species exhibit colony dispersal phenotypes in response to temperature gradients^19^. In *Yersinia pestis*, the pathogen responsible for the plague disease, RNA transcripts encoding for a DGC were found to be degraded at temperatures above 34 °C, thus repressing the production of biofilm-promoting factors^20^. A link between temperature change and c-di-GMP was also established in *Escherichia coli*. A PDE containing a blue light-sensing BLUF domain was found to dimerise and be inactive at low temperatures (~20 °C) while the active monomer form that degrades c-di-GMP was stable at higher temperatures (37 °C)^21^. Finally, temperature shifts were recently shown to affect biofilm formation in the pathogen *Vibrio cholerae*. Decreases in temperature from 37 °C to 25 °C or 15 °C were found to trigger an increase in intracellular levels of c-di-GMP and increased biofilm biomass, and a mutant lacking 6 DGC-encoding genes did not form biofilms in response to a temperature downshift^22^.

In this study, we first explored the effect of temperature variations on biofilms of the opportunistic human pathogen and model organism *Pseudomonas aeruginosa*. We found that increases in temperature can induce the dispersal of pre-established biofilms while temperature decreases resulted in less cells shed from the biofilm. Based on these observations, we then assessed the potential of using iron oxide nanoparticles (IONPs) to induce local hyperthermia and disperse biofilms. IONPs are inorganic nanomaterials with unique physical properties including high surface area to volume ratios and superparamagnetism, which allows them to absorb the energy of an alternating magnetic field and convert it into heat. IONPs have good safety profiles and are commonly used as contrast agents during magnetic resonance imaging (MRI), as well as being investigated for their potential to increase tissue temperature and kill tumour cells by hyperthermia^23^. Due to their high surface energies and capacity to adsorb proteins, IONPs have a tendency to rapidly form aggregates in biological media, which can limit their usage *in vivo*. Coating the IONPs surface with polymers has been shown to stabilize and maintain the nanoparticles in a dispersed state, as well as allowing for addition of multiple functionalities such as conjugation with therapeutic (e.g. antibiotic) or targeting (e.g. antibody) agents and permeation enhancers^24^,^25^. Herein we report the synthesis of block copolymer poly((oligo(ethylene glycol) methyl ether acrylate)-*block*-poly(monoacryloxy ethyl phosphate)-coated IONPs (POEGA-*b*-PMAEP@IONPs) and demonstrate their efficacy at dispersing biofilms in a non-toxic manner while greatly improving the outcome of antibiotic therapies.

## Results

### Temperature upshifts induce biofilm dispersal in *P. aeruginosa*

**Figure 1:**
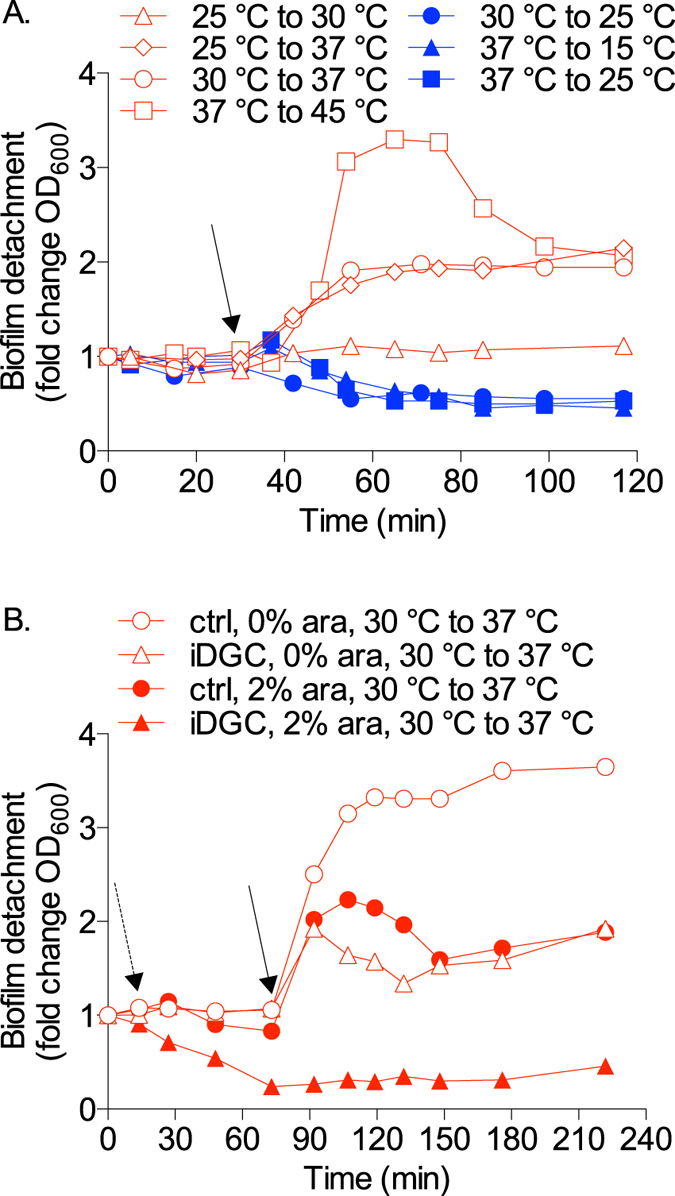
Temperature upshifts induce biofilm dispersal. (**A**) *P. aeruginosa* biofilms were grown in continuous flow microfermentor cultures at 25 °C, 30 °C or 37 °C for 24 h, before switching the temperature. The biofilm effluent was collected at regular intervals and quantified at OD_600_. (**B**) *P. aeruginosa* strains carrying the empty vector pJN105 (ctrl) or pJN105-*wspR* (iDGC) which allows for the arabinose-inducible expression of the DGC WspR were grown in microfermentors for 24 h at 30 °C without arabinose. Then, the biofilms were treated with 2% arabinose (ara) or left untreated for 1 h before increasing the temperature. Solid arrows indicate temperature upshift or downshift, while dashed arrow indicates addition of arabinose. The data shown are representatives from at least 2 (**A**), 25 °C to 30 °C, 37 °C to 45 °C and temperature decreases) or 3 (**A**), 25 °C or 30 °C to 37 °C; (**B**) independent replicate experiments.

The effect of temperature changes on biofilm dispersal was first assessed by using a continuous flow microfermentor culture system^26^. *P. aeruginosa* biofilms were grown for 24 h at 25 °C, 30 °C or 37 °C before the temperature was suddenly changed. In all cases, increasing the temperature by ≥5 °C resulted in the rapid release of dispersal cells in the biofilm effluent, as seen by increases in OD_600_ measurement. A shift by 8 °C from 37 °C to 45 °C gave the strongest dispersal response with a peak showing a 3.3-fold increase in dispersed cells biomass compared to basal detachment levels (Fig. 1A). A shift from 30 °C to 37 °C and 25 °C to 37 °C resulted in 2.0-fold increase in dispersal cells, while a shift from 25 °C to 30 °C only resulted in 10% increase in dispersal. Microscopy analysis of the biofilm effluent confirmed that increases in OD_600_ in the biofilm effluent correlated with an increase in the number of bacterial cells (data not shown). In contrast, when the temperature was reduced from 30 °C to 25 °C or from 37 °C to 25 °C or 15 °C, the amount of biomass released in the effluent appeared to decrease as seen by a reduction of 45% to 55% in OD_600_ measurements, suggesting that basal, non-specific cell detachment was inhibited upon temperature downshift (Fig. 1A). Taken together these data strongly suggest that hyperthermia associated with an increase in temperature is a potent signal for biofilm dispersal.

Because the intracellular messenger c-di-GMP is a master regulator of motile to sessile transitions in bacteria and was already found to be linked to temperature sensing in bacteria^21^,^22^, we then assessed its potential role in regulating temperature-mediated biofilm dispersal. A mutant strain carrying the arabinose-inducible expression vector pJN105-*wspR* (iDGC) was grown in microfermentors and expression of the DGC WspR was induced by adding arabinose 1 h before shifting the temperature. This timing allowed sufficient time to increase intracellular levels of c-di-GMP from DGC activity. Under these conditions that maintained high c-di-GMP levels, the dispersal response to temperature upshift from 30 °C to 37 °C was completely inhibited (Fig. 1B). In the absence of arabinose induction, the iDGC mutant exhibited reduced dispersal compared to the control experiment, which may be explained by leaky activity of the P_bad_ promoter and expression of the DGC WspR in this strain. Addition of arabinose to the vector control strain also appeared to affect the dispersal response, a phenomenon which had been observed in a previous study in *P. aeruginosa* PA14^27^. Next, the potential role of the c-di-GMP-responsive periplasmic protease LapG was assessed. In *Pseudomonas putida* and *Pseudomonas fluorescens* biofilms, starvation was found to trigger a decrease in intracellular c-di-GMP levels which in turn activated LapG to cleave adhesins and lead to surface detachment^28^,^29^. Here, biofilms of a *P. aeruginosa* transposon mutant unable to express *lapG* were found to not disperse in response to a range of cues in microfermentors assays, namely NO signals, O_2_ depletion and starvation (Supplementary information, Fig. S1A), which are all associated with decreases in intracellular c-di-GMP^9^,^30^,^31^. These data confirm that the protease LapG plays a prominent role in the regulation of dispersal in *P. aeruginosa*. Surprisingly, the *lapG* mutant strain was not fully inhibited in the dispersal response to temperature upshift from 30 °C to 37 °C in the microfermentor assay (Supplementary information, Fig. S1B) nor from 37 °C to 45 °C (data not shown), suggesting that biofilm cell detachment in this assay involves other mechanisms than LapG-mediated proteolysis.

### Synthesis and thermal characterization of block copolymer POEGA-*b*-PMAEP-coated IONPs

**Figure 2:**
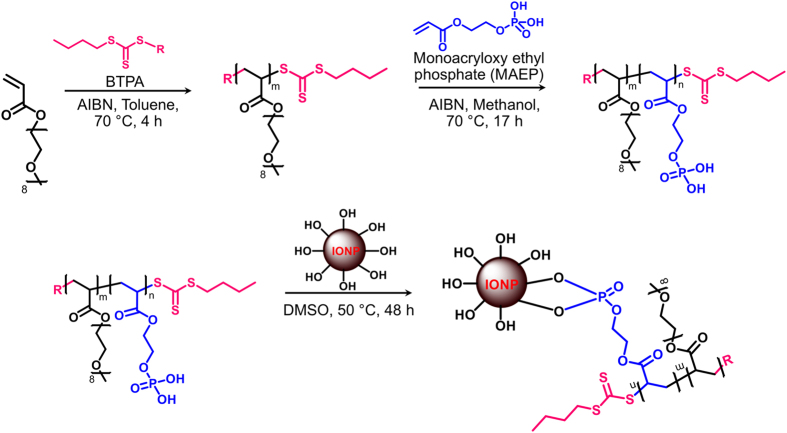
Schematic approach for the preparation of POEGA-*b*-PMAEP@IONPs via RAFT polymerization and “grafting onto” method.

The use of IONPs allows for local control of temperature and inducing hyperthermia. To induce local hyperthermia to biofilms, we made block copolymer POEGA-*b*-PMAEP-stabilized IONPs. First block copolymers POEGA-*b*-PMAEP were synthesized by using living radical polymerization (i.e. reversible addition fragmentation chain transfer (RAFT) polymerization) (Fig. 2). Poly((oligo(ethylene glycol) methyl ether acrylate) (POEGA) macro-RAFT agent was prepared in toluene at 70 °C in the presence of *n*-butyltrithiocarbonate isopropionate (BTPA) as a RAFT agent and oligo(ethylene glycol) methyl ether acrylate (OEGA) as monomer. The conversion of the monomer was determined using ^1^H NMR spectroscopy by comparing the intensity of vinyl proton peaks (6.5 and 5.9 ppm) to that of ester –OCH_2_ proton peaks (δ ~ 4.1 ppm). At ~80% monomer conversion, the polymerization was stopped to avoid the formation of significant dead polymers, and the polymer was purified by several precipitations in a mixture of petroleum ether and diethyl ether (70:30, v/v). The molecular weight of POEGA obtained by SEC analysis was in agreement with the theoretical value (M_*n(theo)*_ = 10,000 g mol^−1^, M_*n(SEC)*_ = 9,400 g mol^−1^, polydispersity index (PDI) = 1.15). POEGA was successfully prepared with low molecular weight distribution and used as a macro-RAFT agent for the polymerization with monoacryloxy ethyl phosphate (MAEP) to afford block copolymer POEGA-*b*-PMAEP. The copolymers were dialyzed against methanol for 48 h to remove unreacted MAEP monomers. After purification, ^1^H and ^31^P NMR spectra confirmed MAEP incorporation by the presence of characteristic signals at ~2.6 ppm and −0.15 ppm, respectively (Supplementary information, Fig. S1). The conversion of the MAEP was determined to be about 50% by using ^1^H NMR spectroscopy and SEC (M_*n(theo)*_ = 11,000 g mol^−1^, M_*n(SEC)*_ = 11,200 g mol^−1^, PDI = 1.18). SEC analysis confirmed that the chains successfully extended and that the molecular weight distribution shifted to higher molecular weight while the polydispersity remained low (PDI < 1.20) (Supplementary information, Fig. S2).

**Figure 3:**
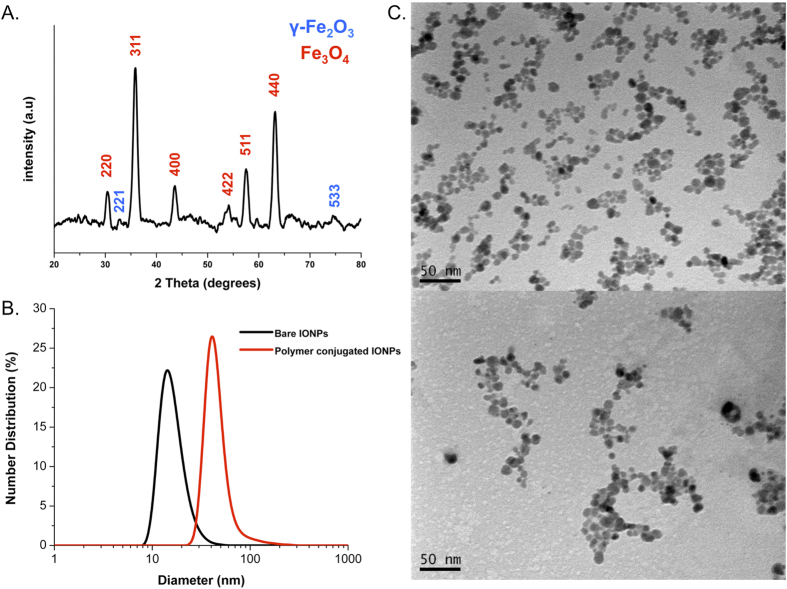
Characterization of POEGA-*b*-PMAEP@IONPs. (**A**) Typical X-ray diffraction (XRD) patterns of bare IONPs. Peaks were indexed according to the *Fd3m* space group. (**B**) Number distribution obtained by dynamic light scattering (DLS) of nanoparticles before and after conjugation with polymer. (**C**) Transmission electron microscopy (TEM) micrographs of dried nanoparticles before (top) and after (bottom) conjugation with polymer.

Next, IONPs were prepared by the co-precipitation method^32^,^33^, yielding a mixture of magnetite Fe_3_O_4_ and maghemite γ-Fe_2_O_3_. Characterization by X-ray diffraction analysis (XRD) revealed a typical pattern corresponding to the spinel structure of γ-Fe_2_O_3_ and Fe_3_O_4_ (Fig. 3A). Then, IONPs were conjugated with the block copolymers. Dynamic light scattering (DLS) analysis revealed that the hydrodynamic diameter of IONPs increased from 16 nm to 44 nm upon polymer grafting (Fig. 3B). The morphology of bare IONPs and polymer-grafted IONPs was also analysed by transmission electron microscopy (TEM), which measures the dried form of the nanoparticles (Fig. 3C). The dried nanoparticles showed a uniformly distributed spherical shape, with a size of about 12 nm, which did not appear to increase after polymer conjugation. Since TEM does not detect the POEGA-*b*-PMAEP polymers, this observation confirms that the increase in size observed by DLS analysis accounted for the successful polymer grafting rather than an increase in size of the IONPs. The combination of DLS and TEM analyses showed the good colloidal stability of the prepared IONPs for biomedical application. The polymer content in the POEGA-*b*-PMAEP@IONPs hybrids was then determined by thermogravimetric analysis (TGA), which measures the weight loss of polymers upon grafting onto metal nanoparticles indicative of the grafting density. A weight loss of about 34% was observed for the polymer-IONPs hybrids, corresponding to a grafting density of approximately 0.25 polymer chain per nm^2^ (Supplementary information, Fig. S3).

**Figure 4:**
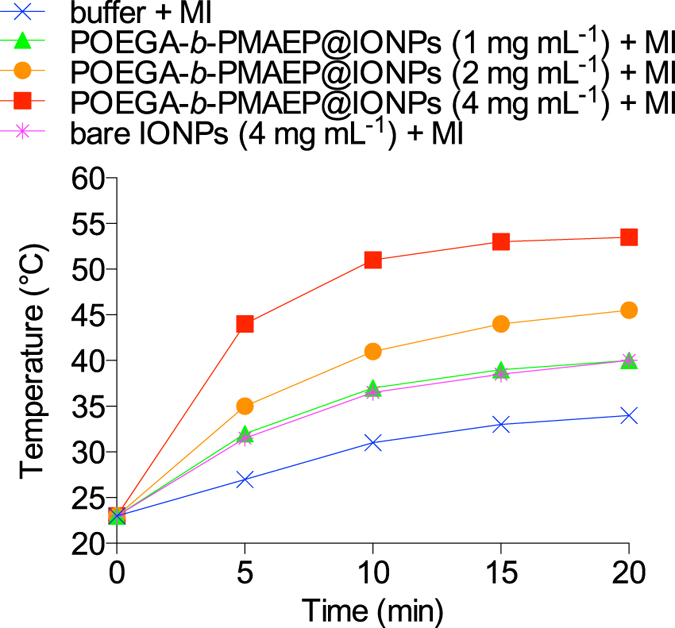
Temperature increases of buffer solutions treated or not with bare IONPs (without polymer) or POEGA-*b*-PMAEP@IONPs and exposed to an alternating magnetic field (6.5 T, 196 kHz) generated with the induction heating system. MI, magnetic induction.

To characterize the thermal properties of IONP-conjugated polymers, bare IONPs or POEGA-*b*-PMAEP@IONPs solutions (1 mL) in phosphate-buffered saline (PBS, pH 7.4) were placed inside the coil of an alternating current (AC) magnetic field generator. Internal or external factors that prevent the IONPs magnetic dipole from following the applied alternating magnetic field result in the conversion of the magnetic energy into dissipated heat^34^. Here we observed a IONP dose-dependent increase in temperature upon magnetic field activation (Fig. 4). Initially, the temperature went up rapidly before reaching a plateau after the first 10 min, regardless of the IONPs concentration. A buffer only solution without any IONP that was used as a negative control, was found to warm up to 34 °C due to heat radiating from the magnetic field generator coil. In the presence of a magnetic field bare IONPs appeared to rapidly aggregate, while POEGA-*b*-PMAEP@IONPs remained well dispersed (data not shown). The non-aggregative behaviour of the polymer-grafted IONPs appeared to increase their heating capacity, as a solution of only 1 mg mL^−1^ POEGA-*b*-PMAEP@IONPs was sufficient to reach 40 °C, while a concentration of 4 mg mL^−1^ of bare IONPs was needed to achieve the same temperature (Fig. 4). When the POEGA-*b*-PMAEP@IONPs concentrations were increased to 2 and 4 mg mL^−1^, the temperatures went up to 46 and 54 °C, respectively. POEGA-*b*-PMAEP@IONPs were used at a final concentration of 1 mg mL^−1^ (iron-based) for all further hyperthermia experiments.

### IONP-induced hyperthermia triggers biofilm dispersal

**Figure 5:**
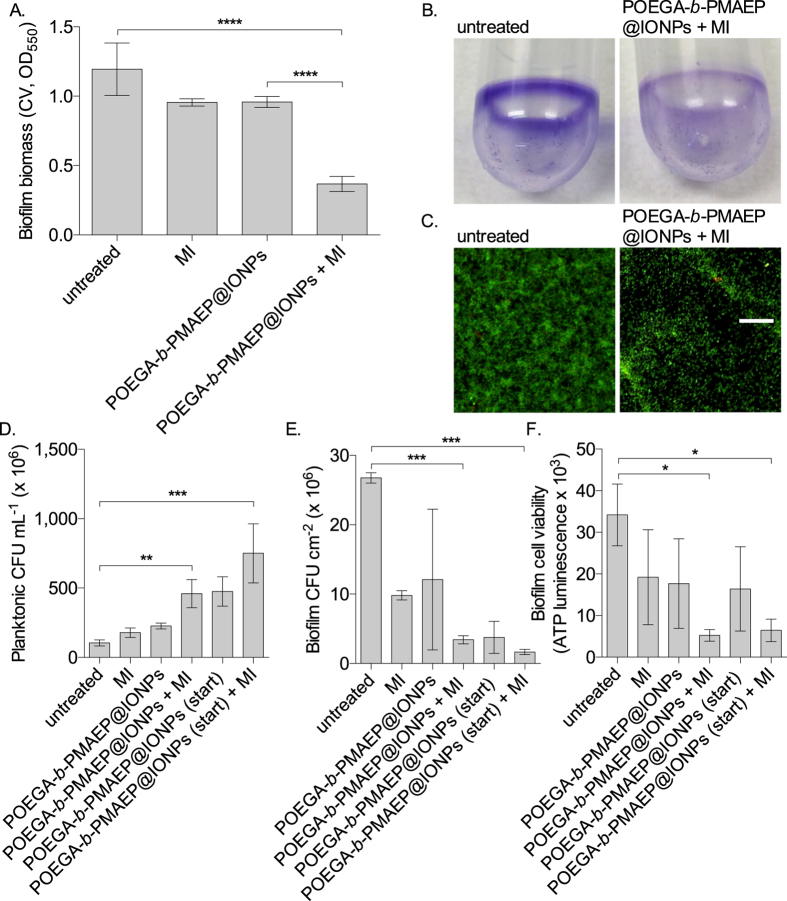
IONP-induced hyperthermia triggers biofilm dispersal in *P. aeruginosa*. (**A**) Biofilms were grown for 24 h in batch culture in test tubes at 23 °C (room temperature). Then, POEGA-*b*-PMAEP@IONPs (1 mg mL^−1^) were added to the cultures and the tubes were immediately exposed (+MI) or not to an alternating magnetic field (6.5 T, 196 kHz) for a further 20 min before analysing biofilms by crystal violet (CV) staining. (**B**) Images of CV stained untreated and treated biofilms. (**C**) Confocal microscopy images of untreated and treated biofilms stained with LIVE/DEAD (live cells appear green, dead cells appear red; bar = 50 μm). (**D**–**F**) After hyperthermia the number of viable cells decreases in the biofilm, but increases in the planktonic phase. Biofilms were grown in test tubes as described above and were treated with POEGA-*b*-PMAEP@IONPs (1 mg mL^−1^) either after 24 h, or from the beginning of growth (start). All induced biofilms were exposed to an alternating magnetic field for 20 min only after the initial 24 h phase of growth. After that time, bacterial viability was assessed by performing colony-forming units (CFU) analyses of the planktonic (**D**) and biofilm (**E**) phases, as well as luciferase-based intracellular ATP measurement of biofilm cells (**F**). MI, magnetic induction. Error bars represent standard errors ((**A**), n ≥ 4; (**D–F**), n = 3). Asterisks indicate statistically significant difference of treatment versus untreated culture (*P < 0.1; **P < 0.01; ***P < 0.001; ****P < 0.0001).

To test the effect of IONP-mediated hyperthermia on biofilm dispersal, biofilms were grown statically in 14 mL tubes at room temperature, a set up that is compatible with the coil of the induction heating system used to activate IONPs. When the biofilms were exposed to magnetic induction only, which increases the temperature from 23 °C to 34 °C, the biofilm biomass was reduced by 20.0% compared to untreated controls (Fig. 5A). Treatments with POEGA-*b*-PMAEP@IONPs without induction also induced a 19.7% decrease in biofilms which may be due to the presence of iron in the nanoparticles. When POEGA-*b*-PMAEP@IONPs were induced with a magnetic field, which leads to an increase in temperature from 23 °C to 40 °C, 69.2% of the biofilm biomass had dispersed after 20 min (Fig. 5A,B). Confocal microscopy analysis of biofilms before and after treatment with magnetically induced POEGA-*b*-PMAEP@IONPs confirmed that biofilm biomass was greatly reduced after 20 min (Fig. 5C). In addition the treatment did not appear to be toxic to bacteria as cells remaining on the surface stained green indicating they were viable bacteria.

Next, the impact of IONPs and IONP-induced hyperthermia on the viability of bacteria was assessed by using colony-forming units (CFU) and intracellular adenosine triphosphate (ATP) analyses. The data show that all treatments resulted in a concomitant increase in planktonic CFU counts and decrease in biofilm CFU counts (Fig. 5D,E), which further confirms that the observed effect are due to a non-toxic dispersal response rather than biofilm reduction via a bactericidal mechanism. Heat induced by magnetic induction only, in the absence of IONP, resulted in a 63.3% decrease in biofilm CFU and 69.8% increase in planktonic CFU compared to untreated controls. Treatment with POEGA-*b*-PMAEP@IONPs whether for 20 min after the initial 24 h of growth, or from the beginning of growth for the entire 24 h incubation did not show any toxicity towards planktonic bacteria and in fact these treatments increased planktonic CFU by 116% and 352%, respectively. The treatment that was most effective at inducing dispersal was when POEGA-*b*-PMAEP@IONPs were added from the start of growth and after 24 h magnetically activated for 20 min, which resulted in a 93.8% reduction in biofilm CFU and 614% increase in planktonic CFU compared to untreated cultures. The trends observed with biofilm CFU analyses were confirmed when performing luciferase-based intracellular ATP measurements of biofilm cells. All treatments resulted in decreased ATP levels, with POEGA-*b*-PMAEP@IONPs added either after or before the 24 h growth incubation, and magnetically activated for 20 min, resulting in 84.6% and 81.1% decrease in ATP, respectively, compared to untreated biofilms (Fig. 5F).

### Mutants affected in the c-di-GMP signalling pathway show impaired dispersal in response to hyperthermia

**Figure 6:**
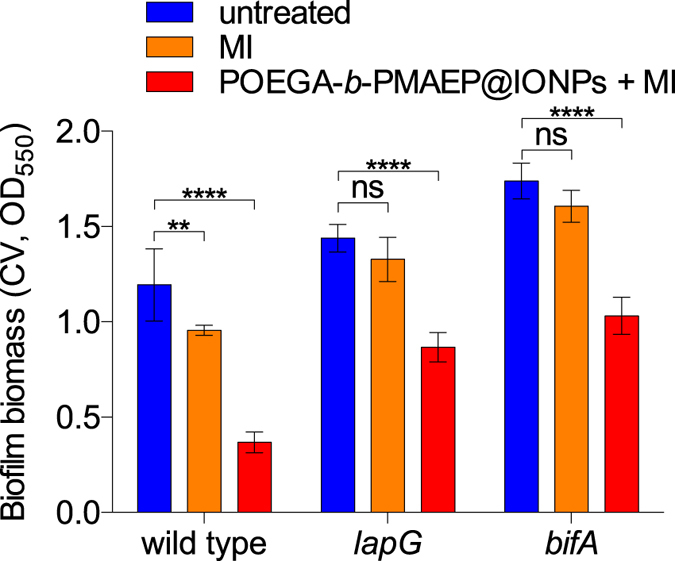
*P. aeruginosa* strains mutated in key c-di-GMP genes show an attenuated response to IONP-induced hyperthermia. Biofilms of *P. aeruginosa* wild type, *lapG* (c-di-GMP responsive periplasmic protease) and *bifA* (c-di-GMP phosphodiesterase) mutant strains were grown in test tubes for 24 h, before being treated with POEGA-*b*-PMAEP@IONPs (1 mg mL^−1^) and exposed to an alternating magnetic field for 20 min, and finally analysed by crystal violet (CV) staining as described before. MI, magnetic induction. Error bars represent standard errors (n = 3). Asterisks indicate statistically significant difference of treatment versus untreated culture (**P < 0.01; ****P < 0.0001; ns, not significant).

Because temperature-mediated dispersal appeared to involve the secondary messenger c-di-GMP, we then tested the effect of hyperthermia and POEGA-*b*-PMAEP@IONPs on biofilms of two mutant strains affected in *lapG* and *bifA*, which are key genes related to c-di-GMP and biofilm dispersal: LapG, as described above is a periplasmic protease that is activated when intracellular c-di-GMP levels decrease, while BifA is a major PDE regulating biofilm development and surface-associated motility in *P. aeruginosa*^35^. Both *lapG* and *bifA* were previously identified in a genetic screen for dispersal deficient mutant biofilms in *P. putida*^36^. While *lapG* was shown to not be solely required for temperature-induced dispersal in microfermentor assays, as described above, in the test tube batch biofilm assay, *lapG* mutant biofilms were only reduced by 7.7% after magnetic induction alone (representing a temperature increase from 23 °C to 34 °C) versus untreated controls, which represent a 61% decrease in the dispersal response compared to wild type biofilms (Fig. 6). Similarly *bifA* mutant biofilms dispersed by only 7.6% in response to magnetic induction. *lapG* and *bifA* biofilms dispersed by 39.8% and 40.7% after magnetic induction in the presence of POEGA-*b*-PMAEP@IONPs, which represent 43% and 41% decrease, respectively, in the dispersal response compared to wild type biofilms (Fig. 6).

### POEGA-*b*-PMAEP@IONPs enhance the efficacy of gentamicin antibiotic therapy

Manipulation of biofilm dispersal regulatory pathways is a promising strategy to develop novel measures against antibiotic resistant biofilms, as it allows for the physical removal of biofilm bacteria in a non-toxic manner by stimulating the cells’ own genetic circuit for dispersal. Once released from the biofilm, dispersal cells are expected to increase their susceptibility to antimicrobial treatments or immune defences. However the release of bacteria that were once attached may cause downstream problems and lead to sepsis or acute infection. One possible approach to circumvent this problem is to combine dispersal-inducing treatments with adjunctive antimicrobial therapies to inactivate the dispersed cells. Here, to assess the potential of POEGA-*b*-PMAEP@IONPs in combinatorial treatments against biofilms, we tested the effect of POEGA-*b*-PMAEP@IONPs together with the commonly prescribed aminoglycoside antibiotic gentamicin on *P. aeruginosa* biofilms.

**Figure 7:**
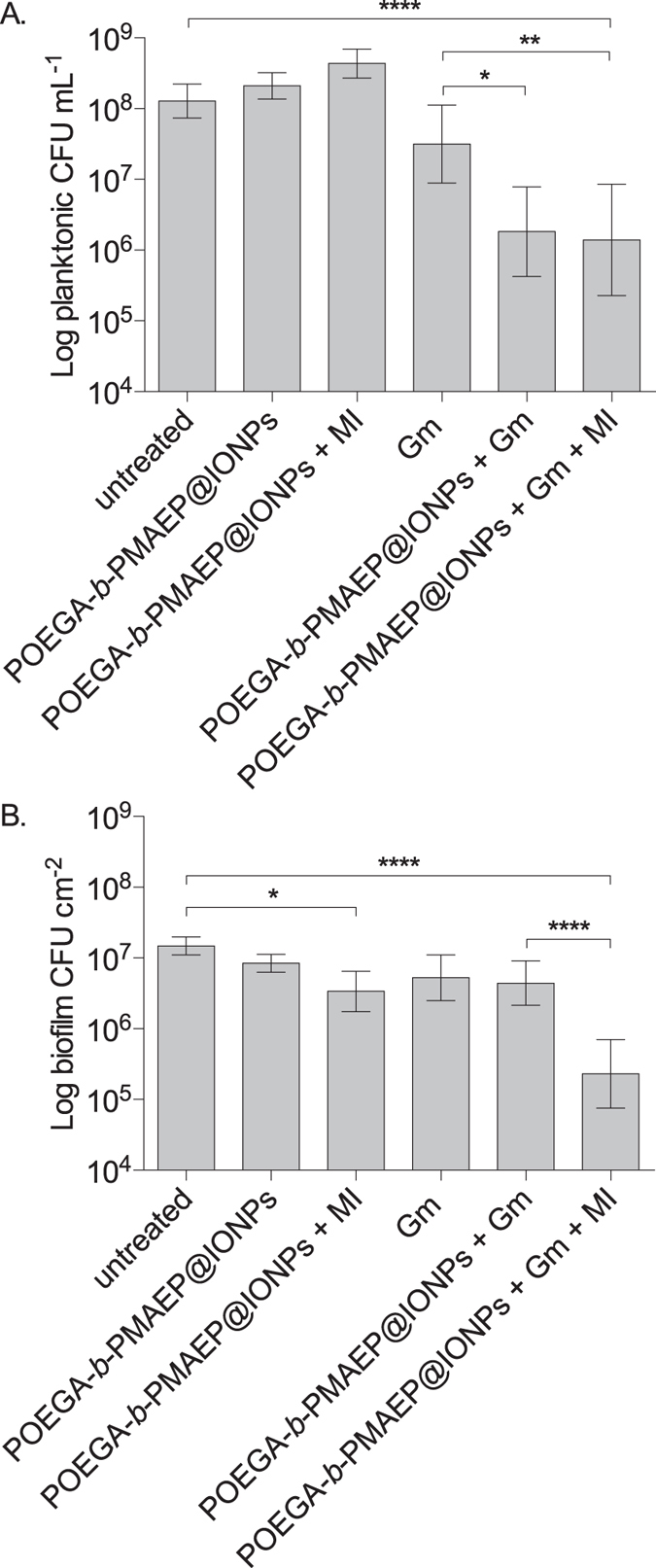
IONP-induced hyperthermia increases the efficacy of the antibiotic gentamicin against both biofilm and planktonic *P. aeruginosa*. Biofilms were grown in test tubes for 24 h before adding POEGA-*b*-PMAEP@IONPs (1 mg mL^−1^), gentamicin (Gm, 2 mg mL^−1^), a combination of both or no treatment to the cultures, and exposing (+MI) or not the cultures to an alternating magnetic field (6.5 T, 196 kHz) for a further 20 min. After treatment, colony-forming units (CFU) analyses were performed of both the planktonic (**A**) and biofilm (**B**) phases. MI, magnetic induction. Error bars represent standard errors (n ≥ 4). Asterisks indicate statistically significant difference of treatment versus untreated culture (*P < 0.1; **P < 0.01; ****P < 0.0001).

In batch biofilm cultures that were treated with gentamicin alone for 20 min, the planktonic phase contained 3.2 × 10^7^ CFU mL^−1^, compared to 1.3 × 10^8^ CFU mL^−1^ in untreated cultures (Fig. 7A). Treatments with activated POEGA-*b*-PMAEP@IONPs alone induced an increase to 4.3 × 10^8^ planktonic CFU mL^−1^, but when combined with gentamicin, the planktonic population was reduced to 1.4 × 10^6^ CFU mL^−1^ after 20 min, which represent a 2-log decrease in CFU compared to untreated samples and a 3.2-fold improvement in the efficacy of gentamicin. Further, when the cultures were treated for 2 h with combined POEGA-*b*-PMAEP@IONPs and gentamicin, planktonic CFU were decreased to 3.6 × 10^3^ CFU mL^−1^, thus a ≥4-log decrease compared to untreated samples (Supplementary information, Fig. S5).

Combined POEGA-*b*-PMAEP@IONPs and gentamicin treatments appeared to be also effective against *P. aeruginosa* biofilms, suggesting that biofilm bacteria that remained on the surface after treatment displayed increased susceptibility towards antimicrobials compared to intact biofilms. Exposure to activated POEGA-*b*-PMAEP@IONPs alone and gentamicin alone for 20 min resulted in 3.2 × 10^6^ biofilm CFU cm^−2^ and 5.3 × 10^6^ biofilm CFU cm^−2^, respectively, compared to 1.3 × 10^7 ^CFU cm^−2^ in untreated biofilms (Fig. 7B). When activated POEGA-*b*-PMAEP@IONPs and gentamicin were added together, biofilm bacteria were reduced to 2.9 × 10^5^ CFU cm^-2^, almost a 2-log decrease compared to untreated biofilms. Treatments with POEGA-*b*-PMAEP@IONPs and gentamicin in the presence of a magnetic field were significantly (*P* < 0.0001) more efficient in reducing biofilm CFU compared to biofilms that were treated with POEGA-*b*-PMAEP@IONPs and gentamicin but not activated with a magnetic field. Taken together these data strongly suggest that inducing hyperthermia with polymer-stabilized IONPs and adjunct antimicrobial therapy is a promising strategy to control biofilms and biofilm-related infections.

## Discussion

The dispersal phase plays critical ecological and evolutionary roles for biofilm populations as it allows for colonization of new habitats and ensures continuity of the species^7^. In this study we reveal that variation in ambient temperature is an important factor regulating dispersal. Temperature is a major determinant for bacterial growth, and the ability to trigger dispersal in response to changes in environmental conditions is likely to optimize chances of survival and successful colonization of new surfaces by dispersal cells. The effect of temperature on biofilm dispersal may not be restricted to *P. aeruginosa* but be conserved across a range of species. In eukaryotes, temperature is well known to be a key factor regulating adaptive physiological changes, for instance by controlling seed germination in plants^37^ and life cycle transitions (e.g. metamorphosis) in animals^18^. Intriguingly, mistletoe plants were recently reported to produce endogenous heat specifically to induce the release and dispersal of seeds from ripe fruits^38^, a process reminiscent of the release of single cells from mature biofilms, which has been referred to as seeding dispersal^7^. Temperature-induced dispersal may also be important in host-microbe interactions and immune responses to pathogen infections, where increasing tissue temperature, e.g. by triggering fever, could contribute to clearing bacterial infections by inducing detachment events and preventing the formation of a persistent biofilm.

Our data suggest that temperature upshifts trigger a signalling pathway involving the secondary messenger c-di-GMP in *P. aeruginosa*. Several reports in the literature have already identified a link between temperature and c-di-GMP, whereby higher temperatures were associated with decreased c-di-GMP levels and biofilm formation in *Y. pestis* and *E. coli*^20^,^21^, or whereby temperature downshifts were found to trigger biofilm formation in *V. cholerae*^22^. These studies are consistent with the observations reported here that temperature negatively regulates biofilm formation, suggesting that these effects may be conserved across species. The signalling pathway from temperature sensing to dispersal remains to be fully elucidated. The periplasmic protease LapG appears to play a role in regulating dispersal in batch cultures although the transposon mutant strain was not fully impaired in the dispersal response (Fig. 6), suggesting that other mechanisms may be involved in inducing surface detachment. Furthermore, the *lapG* mutant was not strongly affected in dispersal in continuous flow cultures (Supplementary information, Fig. S1), which may be due to differential expression of c-di-GMP associated genes particularly those interacting with LapG and its cognate c-di-GMP sensor LapD^39^,^40^ in microfermentor-grown biofilms compared to batch culture biofilms. The PDE BifA also seemed to be involved in regulating dispersal in batch biofilm cultures, but the response was not fully inhibited in the mutant strain (Fig. 6), indicating that other mechanisms such as multiple or redundant PDEs are likely to play a role in this system. Regulation of cellular processes by temperature may occur at different levels, either indirectly via changes in metabolic processes, or directly by affecting enzymatic activity of target enzymes, modulating cell membrane fluidity^22^ or via the stimulation of specific thermosensor proteins capable of interacting with DGC or PDE. Several thermosensors have already been characterized in bacteria, including RNA thermometers involved in cold and heat shock responses, such as CspA in *E. coli*^41^, and the two-component system DesKR in *Bacillus subtilis* that responds to alterations in membrane fluidity caused by temperature changes^42^.

The discovery of a role for temperature in regulating dispersal opens new avenues for biofilm control. Here we used IONPs for the induction of local hyperthermia. First, IONPs were conjugated to block copolymers that allow for enhanced stability and functionalization. Previously, POEGA-*block*-poly(dimethylaminoethyl acrylate) IONPs (POEGA-*b*-PDMAEA@IONPs) were synthesised that did not show any cytotoxicity^43^, here we used MAEP instead of dimethylaminoethyl acrylate to add more phosphonic acid moieties to bind metal oxides and thus improve adhesion and stability of the block copolymers on the IONPs. The new IONP polymers were found to be highly stable and effectively disperse established biofilms. Moreover, both cells remaining on the surface as well as dispersal cells displayed increased susceptibility to gentamicin treatments. Interestingly, enhanced efficacy of a range of antibiotics with different modes of action was already observed in *Staphylococcus aureus* biofilms when grown at 40 °C and 45 °C compared to 35 °C^44^. This suggests that hyperthermia induced by IONP polymers may also be effective with other antimicrobial agents. In fact, strategies to induce biofilm dispersal, e.g. by using low dose NO donor compounds^45^ or availability of nutrients^46^, have already been shown to restore the sensitivity of biofilm cells towards a range of antimicrobials and antibiotics rather than induce a synergistic effect with one specific antibiotic. IONPs had already been studied for their impact on bacterial biofilms. However, in contrast to all previous studies which investigated a toxic, bactericidal effect of IONPs against planktonic or biofilm bacteria^47^,^48^,^49^,^50^, here POEGA-*b*-PMAEP@IONPs both in the activated and non-activated states were non-toxic and in fact, as a result of inducing dispersal events, led to an increase in viable planktonic cells (Figs 5D and 7A). Non-toxic therapies to control microbial communities are much preferred, as they are not expected to select for resistant bacterial cells.

In this work, high concentrations of IONPs as well as high magnetic fields were used in order to induce a sufficient increase in temperature. This technology may be directly appropriate for the development of materials with antibiofilm properties that could be remotely activated by applying a magnetic field. For instance, IONP polymers may be applied as surface coatings, for instance via thermal or solvent deposition which would not affect their performance, that would be suitable for the treatment and disinfection of indwelling medical devices such as catheters, or prosthetic implants which are major sources of biofilm-related contaminations in clinical settings^51^. IONPs may also find broad applications in industrial settings, for example for the disinfection of heat exchanger systems, water distribution pipelines or filtration membranes^52^. In the future, new materials may be designed with enhanced functionalities, e.g. targeted release of antibiotics, optimised for administration in humans to treat infectious diseases, and this work may benefit from recent translational research progress to develop IONPs suitable to induce hyperthermia targeted to tumour cells^53^.

## Methods

### Bacterial strains, culture media and chemicals

The Gram-negative laboratory strain *P. aeruginosa* PAO1 was used to characterize the effects of temperature variations on biofilm formation and dispersal. *P. aeruginosa* PAO1 strains carrying the expression vector pJN105-*wspR* (iDGC), allowing expression of the DGC WspR under control of the arabinose-inducible P_bad_ promoter (Gm^R^), as well as pJN105 vector control (ctrl) were generously provided by Joana Moscoso/Alain Filloux^54^. *P. aeruginosa* mutant strains containing a transposon Tn5-derived insertion element (Tc^R^) in key genes involved in c-di-GMP signalling and biofilm dispersal, *bifA* (PA4367) and *lapG* (PA1434), were obtained from the University of Washington *P. aeruginosa* mutant library: strains PW8371 *bifA*-D09::IS*lacZ*/hah and PW3606 *lapG*-D03::IS*lacZ*/hah, respectively^55^. Overnight cultures were routinely grown in LB-Miller broth with 10 g L^−1^ NaCl with shaking at 37 °C. All chemicals were purchased from Ajax and Sigma-Aldrich, unless otherwise specified. Monomer oligo(ethylene glycol) methyl ether acrylate with an average Mn of 480 g mol^−1^ (OEGA) and monoacryloxy ethyl phosphate (MAEP, Polyscience, Inc.) were used as received. 2,2′-Azobisiobutylronitrile (AIBN) was purified by recrystallization from methanol and stored at 0 °C until required for further use.

### Microfermentor continuous flow biofilm cultures

*P. aeruginosa* biofilms were grown under continuous flow-through and aerated conditions in glass microfermentors, as previously described^26^ with some slight modifications. The microfermentors received a continuous flow of fresh M9 minimal medium (containing 48 mM Na_2_HPO_4_, 22 mM KH_2_PO_4_, 9 mM NaCl, 19 mM NH_4_Cl, 2 mM MgSO_4_, 100 μM CaCl_2_, pH 7.0) with 0.04% glucose and were immersed in a circulating water bath with a thermostat set at 25 °C, 30 °C or 37 °C. After 24 h of growth, cells released from the biofilm were monitored by collecting the effluent runoff approximately every 10 min and measuring the OD_600_. The OD_600_ value of the biofilm effluent measured before inducing dispersal was stable and indicated basal, non-specific shedding of bacterial cells from the biofilm. Temperature upshifts were achieved by increasing the thermostat from 25 °C to 30 °C or 37 °C, from 30 °C to 37 °C, or from 37 °C to 45 °C. For these procedures the temperature in the water bath adjusted within 1 to 3 min. For temperature downshifts, the microfermentors were carefully transferred to another water bath pre-set at 15 °C, 25 °C or 30 °C without disrupting the medium or airflow in the microfermentors. This procedure ensured minimal physical disruption of the biofilms growing inside the microfermentors.

To test the effect of DGC expression on temperature-induced dispersal, biofilms of *P. aeruginosa* carrying pJN105 and pJN105-*wspR* plasmids were grown in microfermentors as described above in M9 medium with 0.04% glucose and 50 μg mL^−1^ gentamicin and without any arabinose for 24 h at 30 °C. Arabinose was added to 2% (w/v) in the biofilm medium reservoir 1 h before increasing the temperature of the water bath to 37 °C. To validate and test the *lapG* mutant that is affected in dispersal to a range of signals and cues, biofilms of *P. aeruginosa* wild type and *lapG* transposon mutant were grown in microfermentors as described above for 24 h at 37 °C before inducing dispersal by suddenly: (i) adding the NO donor sodium nitroprusside (1 mM) to the biofilm medium; (ii) switching the microfermentor aeration from air to 100% nitrogen (N_2_) gas to induce O_2_ depletion; or (iii) switching the biofilm medium to M9 medium without carbon source (no glucose) to induce starvation; or were grown for 24 h at 30 °C before increasing the temperature to 37 °C.

For these experiments similar results were observed in at least two or three independent assays, as indicated. The OD_600_ of the biofilm effluent were normalised to the value before inducing dispersal (time t = 0) for each biofilm, which typically had a value between 0.05 and 0.15.

### Polymer and nanoparticle characterization

#### NMR spectroscopy

^1^H and ^31^P NMR spectra were recorded using a Bruker Avance 300 (300 MHz) spectrometer. CDCl_3_ and MeOD were used as solvents. All chemical shifts are reported in parts per million (ppm) relative to tetramethylsilane (TMS), referenced to the residual solvent frequencies ^1^H NMR: CDCl_3_ = 7.26, MeOD = 3.31 ppm. For ^31^P NMR spectra, ^31^P resonances were externally referenced to 85% H_3_PO4 in D_2_O at 0.00 ppm. The monomer (i.e. OEGA) conversion was calculated by the following equation, where *I*^5.9 ppm^ and *I*^4.2 ppm^ correspond to the integral of the vinyl signal from the monomer at 5.9 ppm (^1^H mol^−1^) and the ester signal from the monomer/polymer at 4.2 ppm (^2^H mol^−1^), respectively.


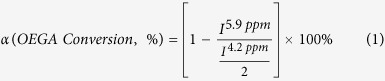


The theoretical molecular weight was calculated by the following equation below:


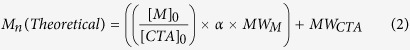



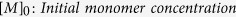



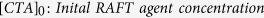



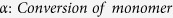



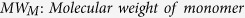



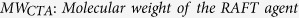


#### Size exclusion chromatography (SEC)

SEC analyses of polymer samples were performed in *N,N*′-dimethylacetamide [DMAc with 0.03% w/v LiBr and 0.05% 2,6-di-butyl-4-methylphenol (BHT)] at 50 °C (flow rate of 1 mL min^−1^) with a Shimadzu modular system comprising and SIL-10AD automatic injector, a Polymer Laboratories 5.0 μL bead-size guard column (50 × 7.8 mm) followed by four linear PL (Styragel) columns (105, 104, 103 and 500 Å) and an RID-10A differential refractive-index detector. The SEC calibration was performed with narrow-polydispersity polystyrene standards ranging between 104 and 2,000,000 g mol^−1^. Polymer solutions at 2–3 mg mL^−1^ were prepared in the eluent and filtered through 0.45 μm filter prior to injection.

#### Dynamic light scattering (DLS)

DLS measurements were performed using a Malvern Zetasizer Nano Series running DTS software (4 mW, He-Ne laser, λ = 633 nm) and an avalanche photodiode (APD) detector. The scattered light was measured at an angle of 175° for DLS measurements. The temperature was stabilized to ±0.1 °C of the set temperature. All samples were prepared in Milli-Q water at the concentration of ~0.2 mg mL^−1^ of polymer and filtered through a 0.45 μm pore size filter to remove dust prior to measurement.

#### Transmission electron microscopy (TEM)

Nanoparticles size and morphologies were measured and analysed using a JEOL 1400 transmission electron microscope at an accelerating voltage of 80 kV. A drop of samples solution was deposited onto a formvar-coated copper grid and the water was evaporated under air. No staining was applied.

#### Thermogravimetric analysis (TGA)

TGA of IONPs was performed on a Perkin-Elmer Thermogravimetric Analyzer (Pyrus 1 TGA). Pre-dried samples were heated from room temperature to 150 °C at a constant rate of 20 °C min^−1^ using air as the furnace gas. The temperature of 100 °C was kept constant for 30 min to remove moisture from samples. Then, the temperature was returned to 25 °C and heated again to 800 °C at a rate of 20 °C min^−1^. The weight loss was calculated through the difference between the weights at room temperature and at 800 °C. The grafting density was estimated using the weight loss (loss-wt) and the surface area of IONPs determined by BET according to the following equation:


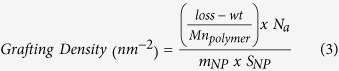


Mn_polymer_ corresponds to the weight of polymer grafted. *N*_a_ is Avogadro’s number and *m*_*NP*_ is the mass of polymer-grafted iron oxide nanoparticles used for the TGA analysis (i.e. mass of nanoparticles = initial mass before TGA analysis – loss of weight).

#### X-ray diffraction (XRD)

The crystal characterization of IONPs was performed using a Philips PANanalytical Xpert X-ray Multipurpose Diffraction System at 40 mA and 45 kV employing monochromated Cu-Ka radiation (λ = 1.541 Ǻ, step size = 0.01, 0.02 or 0.05, time per step = 10 or 20 s/step).

### POEGA-*b*-PMAEP@IONPs synthesis

#### Synthesis of block copolymers POEGA-b-PMAEP

The RAFT agent, 2-(((butylsulfanyl)carbonothioyl)sulfanyl)propanoic acid (BTPA) was prepared according to Ferguson *et al.*^56^. OEGA (10.08 g, 0.021 mol), BTPA (0.1925 g, 0.0008 mol) and AIBN (0.0265 g, 1.6 × 10^−4 ^mol) were dissolved in 25 mL of toluene in a round bottom flask equipped with a magnetic stirrer bar. The flask was sealed with a rubber septum and the reaction mixture was degassed with N_2_ gas under ice for 30 min. Then, the degassed solution was placed in a preheated oil bath at 70 °C and the polymerization was carried out for 4 h. The reaction was terminated by quenching the sample in an ice bath for 15 min and an aliquot was taken for NMR analysis. The conversion of monomer during the course of polymerization was determined using ^1^H NMR. The POEGA polymer was purified by precipitating with excess mixture of petroleum ether and diethyl ether (70:30, v/v) followed by centrifugation (7,500 rpm for 5 min). The precipitation and centrifugation steps were repeated up to four times to remove any traces of unreacted monomer, and the polymer was dried in a vacuum oven (40 °C) overnight to remove the remaining solvent. The purified POEGA was characterized by ^1^H NMR and SEC, and stored at 4 °C until required for further chain extension.

POEGA was used as a macro-RAFT agent for chain extension with MAEP to introduce the functional phosphonic acid group that allows the conjugation to IONPs. POEGA _Mn = 10,000 g mol^−1^_(1.0 g, 1 × 10^−4^ mol), MAEP (0.1961 g, 1 × 10^−3^ mol) and AIBN (0.0016 g, 1 × 10^−5^ mol) were dissolved in methanol (3.5 mL) in a cospak bottle, equipped with a magnetic stirrer bar. The reaction mixture was degassed with N_2_ gas for 30 min in an ice bath. The polymerization was carried out in a preheated oil bath at 70 °C overnight. The reaction was terminated by placing the sample in an ice bath for 15 min and an aliquot was taken for NMR analysis. The conversion of monomer during the course of polymerization was determined using ^1^H NMR. The copolymer was purified by dialysis against methanol for 48 h to remove unreacted MAEP, and the purified polymer was then dried in a vacuum oven overnight to remove remaining solvent. The purified copolymer POEGA-*b*-PMAEP was characterized by ^1^H NMR, ^31^P NMR and SEC, before being stored at 4 °C until required for further use.

#### Synthesis of IONPs

IONPs were prepared via chemical co-precipitation method adapted from Jain *et al.*^33^ with slight modifications. Firstly, 80 mL of 2 M FeCl_3_.6H_2_O (0.16 mol, 43.2 g) in 1 M HCl and 40 mL of 2 M FeCl_2_.4H_2_O (0.08 mol, 15.9 g) in 1 M HCl were mixed in a 2 L beaker, and the mixture diluted to 1.2 L deionized water. A total of 250 mL of ammonium hydroxide (28%) was quickly added into the solution of iron chloride, and the mixture was vigorously stirred for 30 min. The colour of the solution immediately changed to black due to the formation of magnetite. The precipitate was collected using a magnet, the supernatant was discarded and the black solid was re-dispersed in a volume of 293 mL HNO_3_ (2 M) by stirring for 5 min. Then, an equal volume (i.e. 293 mL) of Fe(NO_3_)_3_.9H_2_O (0.35 M) was added to the magnetite dispersion, followed by heating at 90 °C for about 1 h to form maghemite. The precipitate was magnetically collected and then washed twice with 195 mL HNO_3_ (2 M) and twice with 250 mL acetone. Finally, the nanoparticles were dispersed and stored in deionized water which pH was adjusted to about 1.5–2, resulting in a highly stable dispersion. The synthesized magnetic nanoparticles were characterized by several techniques, including XRD, TGA, TEM and DSL.

#### Conjugation of block copolymers POEGA-b-PMAEP to IONPs using “grafting onto” approach

Grafting of phosphonic acid bearing copolymer POEGA-*b*-PMAEP on the surface of IONPs was performed as previously described by our group^57^ with some modifications. Briefly, 10 mg IONPs were dispersed in 4 mL DMSO and the solution was sonicated for 5 min. A solution of 0.0075 mmol of phosphonic acid bearing polymer in 1 mL DMSO was added dropwise to the dispersion of IONPs. The mixture of polymer and IONPs was sonicated for 3 min, followed by incubation at 50 °C with shaking at 100 rpm for 48 h. Upon completion, the solution was filtered by passing through a 0.45 μm pore size filter to remove the unstabilized nanoparticles and then purified by centrifugation (using the Eppendorf Centrifuge 5804) at 10,000 rpm for 30 min. The functionalized IONPs were isolated at the base of the centrifuge tube. The supernatant was removed and the particles were re-dispersed in acetonitrile. The washing process was repeated twice to give polymer functionalized IONPs synthesized via “grafting onto” method. The purified hybrid product was characterized by TGA, DLS and TEM techniques for grafting efficiency, size and morphology, respectively.

### Heat induction derived from the hybrid polymer-IONPs by exposing to an alternating current (AC) magnetic field

Solutions of bare IONPs and POEGA-*b*-PMAEP@IONPs prepared in PBS (1 mL) at various relative iron concentrations were added to 14-mL round bottom polypropylene tubes (Greiner). IONPs activation was achieved by using a EasyHeat induction heating system (Ambrell), equipped with a helical coil, 25 mm internal diameter, 60 mm long with 8 turns. The IONP-containing tube was placed in the centre of the coil and a glass alcohol thermometer was immersed in the solution for temperature measurement. The optimal IONP heating conditions were obtained after 20 min incubation to an alternating magnetic field at a frequency of 196 kHz, which was within the physiological limit of 200 kHz of the human tolerance threshold^58^, and constant 200 A intensity, which generates a magnetic field of about 6.5 T in the centre of the coil.

### Biofilm dispersal with IONP polymers and eradication with antibiotic-IONP combinatorial treatments

To characterize the effects of IONP polymers on biofilm dispersal, *P. aeruginosa* biofilms were grown at 23 °C (room temperature) in batch cultures in individual test tubes under static conditions. Briefly in all assays, overnight cultures in LB-Miller broth were diluted to an OD_600_ of 0.005 in 1 mL M9 minimal medium with 0.4% glucose in 14-mL round bottom polypropylene tubes (Greiner). The tubes were incubated at 23 °C statically and the biofilms were allowed to grow for 24 h without any disruption.

IONP polymers were added to the tubes at different concentrations as indicated either after 24 h incubation or from the beginning of growth. Each treatment was added from a 10 μL aliquot of a stock solution at the appropriate concentration of the compound dissolved in PBS. For antibiotic-IONP combinatorial treatments the aminoglycoside gentamicin sulphate was used at a final concentration of 2 mg mL^−1^ and added to the cultures either alone or together with POEGA-*b*-PMAEP@IONPs. After adding the treatments, the tubes were carefully transferred to a mock location on the bench (no magnetic induction) or to the centre of the coil of the EasyHeat induction heating system operating at constant 200 A intensity and 196 kHz frequency and incubated there for a further 20 min before quantifying the biomass or viability of both planktonic and biofilm bacteria. For combinatorial treatments with POEGA-*b*-PMAEP@IONPs and gentamicin, biofilms to which the treatments had been added were exposed to magnetic induction or left at a mock location for either 20 min or for 2 h.

Biofilm biomass was quantified by using the crystal violet staining method as previously described^26^. Briefly, after incubation the culture supernatant was removed and the biofilm on the tube surfaces was first washed once with PBS (1 mL), before adding 0.03% crystal violet stain (1 mL). The tube was incubated on the bench for 20 min before removing the dye and washing the internal surfaces twice with PBS. Photographs of the stained biofilms were obtained using a digital camera. The amount of remaining crystal violet-stained biofilm was quantified by adding 100% ethanol (1 mL) and measuring the OD_550_ of the homogenized suspension by using a microtitre plate reader (Wallac Victor^2^, Perkin-Elmer). OD measurements of control tubes where no bacteria were added at the beginning of the experiment were subtracted from all values (i.e. OD_550_ = 0.10).

To determine bacterial viability, planktonic and biofilm colony-forming units (CFU) analyses were determined by a drop plate method^59^. For planktonic analysis, free-floating cells in the biofilm supernatant were serially diluted in sterile PBS and plated onto LB agar. For biofilm analysis, cells attached on the interior surfaces of the tubes (surface area 3.8 cm^2^) were washed twice with sterile PBS to remove loosely attached bacteria, before being resuspended and homogenised in PBS by incubating in a sonication bath (150 W, Unisonics) for 20 min^60^. Then, cells were serially diluted and plated onto LB agar. CFU were enumerated after 24 h incubation at 37 °C. The BacTiter-Glo Microbial Cell Viability Assay (Promega, Alexandria, Australia) was also used, which is based on quantitation of the ATP present in bacteria by using a thermostable luciferase. After the final 20 min incubation with various treatments and magnetic induction, biofilm cells were resuspended and homogenized in PBS as described above. Resuspended biofilm cells were mixed with BacTiter-Glo reagent and after 5 min the luminescence was measured by using a multimode microtitre plate reader (Wallac Victor^2^, Perkin-Elmer).

All assays included at minimum 2 replicates and were repeated in at least 2 or 3 independent experiments. Data analysis was performed using means (crystal violet, ATP measurements) or geometric means (CFU) and compared among groups via one-way ANOVA, followed by Tukey’s post test for individual comparisons. Differences were deemed statistically significant at a *P* value < 0.1.

### Confocal microscopy analysis

For microscopy analysis, narrow glass coverslips (10.5 × 35 mm, ProSciTech, Townsville, Australia) were inserted in the biofilm culture tubes and *P. aeruginosa* biofilms were grown otherwise as described above. After 24 h incubation and 20 min treatment, the coverslips harbouring biofilms were carefully removed from the tubes by using sterile tweezers and rinsed twice with PBS before wiping clean one side and staining the other side with LIVE/DEAD *Bac*Light bacterial viability kit reagents (Molecular Probes) according to the manufacturer’s procedure. Three hundred microliters of the staining solution (1:1,000 dilution of each SYTO9 and propidium iodide components in PBS) was trapped between the biofilm sample on the coverslip and a standard microscope slide. After 20 min incubation at room temperature in the dark, the samples were observed with an Olympus FV1000 Confocal Inverted Microscope, and imaged with Leica DFC 480 camera. Cells that were stained green were considered to be viable, those that stained red were considered to be dead.

## Additional Information

**How to cite this article**: Nguyen, T.-K. *et al.* Iron oxide nanoparticle-mediated hyperthermia stimulates dispersal in bacterial biofilms and enhances antibiotic efficacy. *Sci. Rep.* **5**, 18385; doi: 10.1038/srep18385 (2015).

## Supplementary Material

Supplementary Information
