# Supplementary material for: Iron oxide nanoparticle-mediated hyperthermia stimulates dispersal in bacterial biofilms and enhances antibiotic efficacy
Source: Sci Rep. 2015 Dec 18;5:18385. doi: 10.1038/srep18385 (PMC4683393; doi:10.1038/srep18385)
Supplement: Supplementary Information [file srep18385-s1.pdf]

Supplementary Information

Iron oxide nanoparticle-mediated hyperthermia stimulates dispersal in bacterial biofilms and enhances antibiotic efficacy

Thuy-Khanh Nguyen[1], Hien T. T. Duong[1#], Ramona Selvanayagam[1], Cyrille Boyer[1*] & Nicolas Barraud[2†*]

[1]Centre for Advanced Macromolecular Design (CAMD) and Australian Centre for NanoMedicine (ACN), School of Chemical Engineering, The University of New South Wales, Sydney 2052, Australia

[2]Centre for Marine Bio-Innovation, School of Biotechnology and Biomolecular Sciences, The University of New South Wales, Sydney 2052, Australia

[#]Present address: School of Chemistry, The University of Sydney, Sydney 2006, Australia

[†]Present address: Department of Microbiology, Genetics of Biofilms Unit, Institut Pasteur, Paris 75015, France

*These authors contributed equally to this work.

Correspondence and requests for materials should be addressed to C.B. (email: cboyer@unsw.edu.au) or N.B. (email: n.barraud@melix.org).

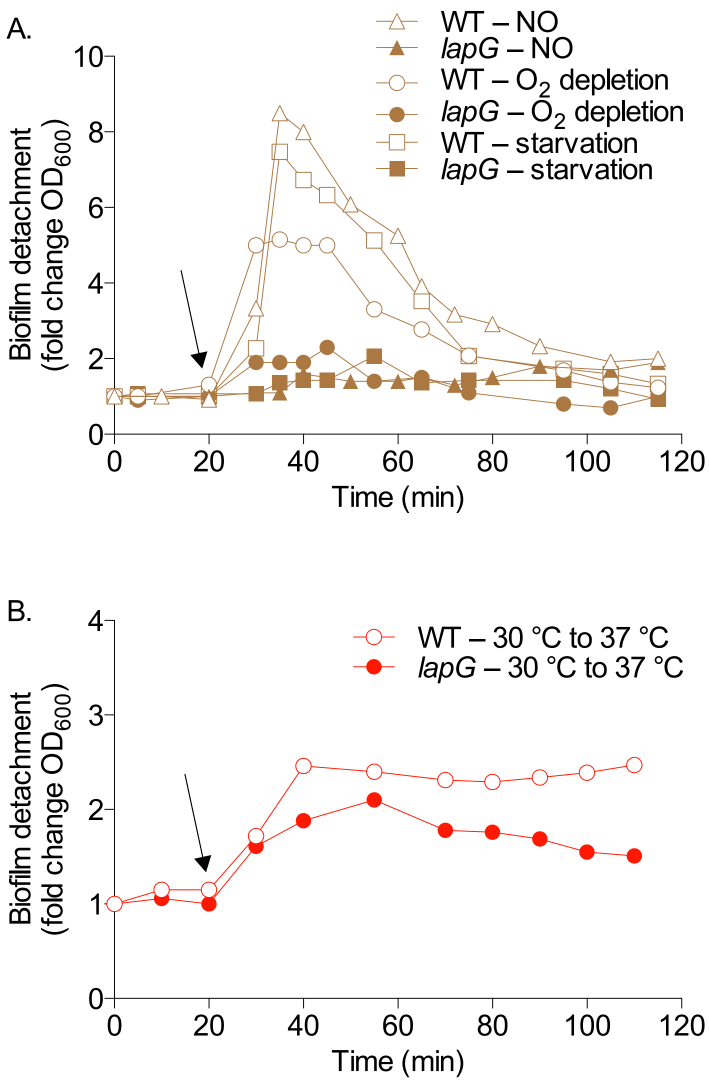

Fig. S1. A *P. aeruginosa lapG* mutant strain, which does not disperse in response to a range of signals and cues, is not strongly affected in temperature-mediated dispersal. (A) *P. aeruginosa* wild type (WT) and *lapG* mutant biofilms were grown in continuous flow microfermentor cultures at 37 °C for 24 h. At this time, biofilm dispersal was induced by: (i) adding the nitric oxide (NO) donor sodium nitroprusside (1 mM) to the biofilm medium; (ii) switching the microfermentor aeration from air to 100% nitrogen gas to induce oxygen (O_2) depletion; or (iii) switching the biofilm medium to M9 medium without carbon source to induce starvation. The biofilm effluent was collected at regular intervals and quantified at OD_{600}. Solid arrows indicate induction of dispersal. (B) *P. aeruginosa* wild type (WT) and *lapG* mutant biofilms were grown in continuous flow microfermentor cultures for 24 h at 30 °C before suddenly increasing the temperature to 37 °C. Solid arrows indicate temperature upshift. The data shown are representatives from at least 3 independent replicate experiments.

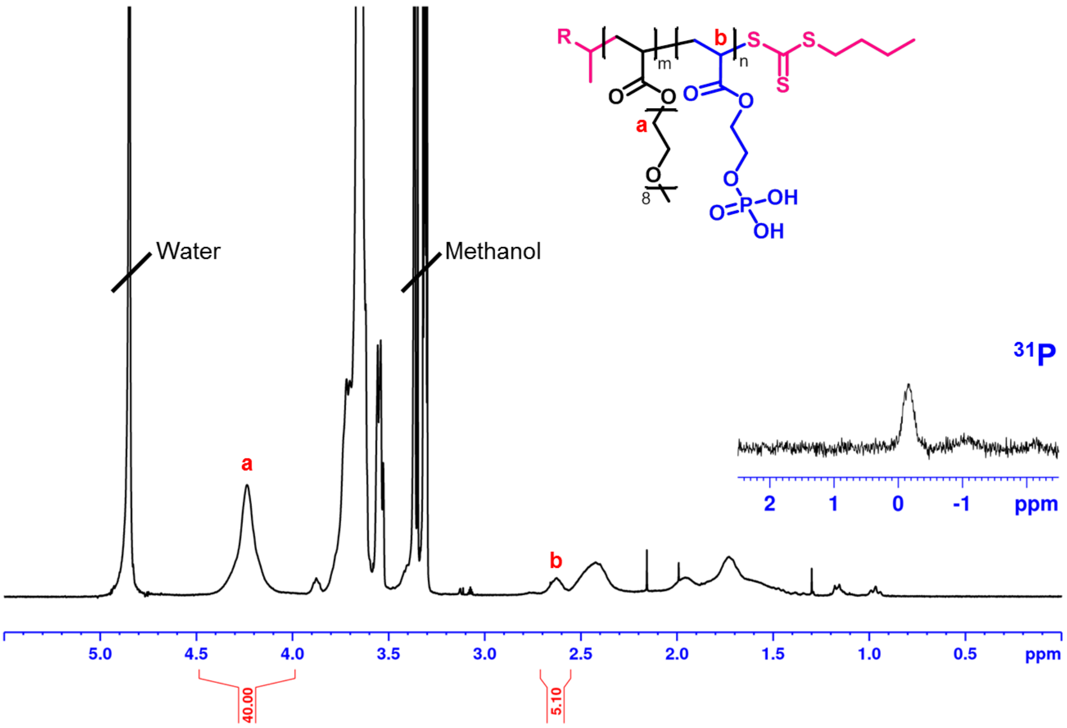

Fig. S2. ^{1}H and ^{31}P spectra of purified POEGA-*b*-PMAEP (recorded in CD_3OD).

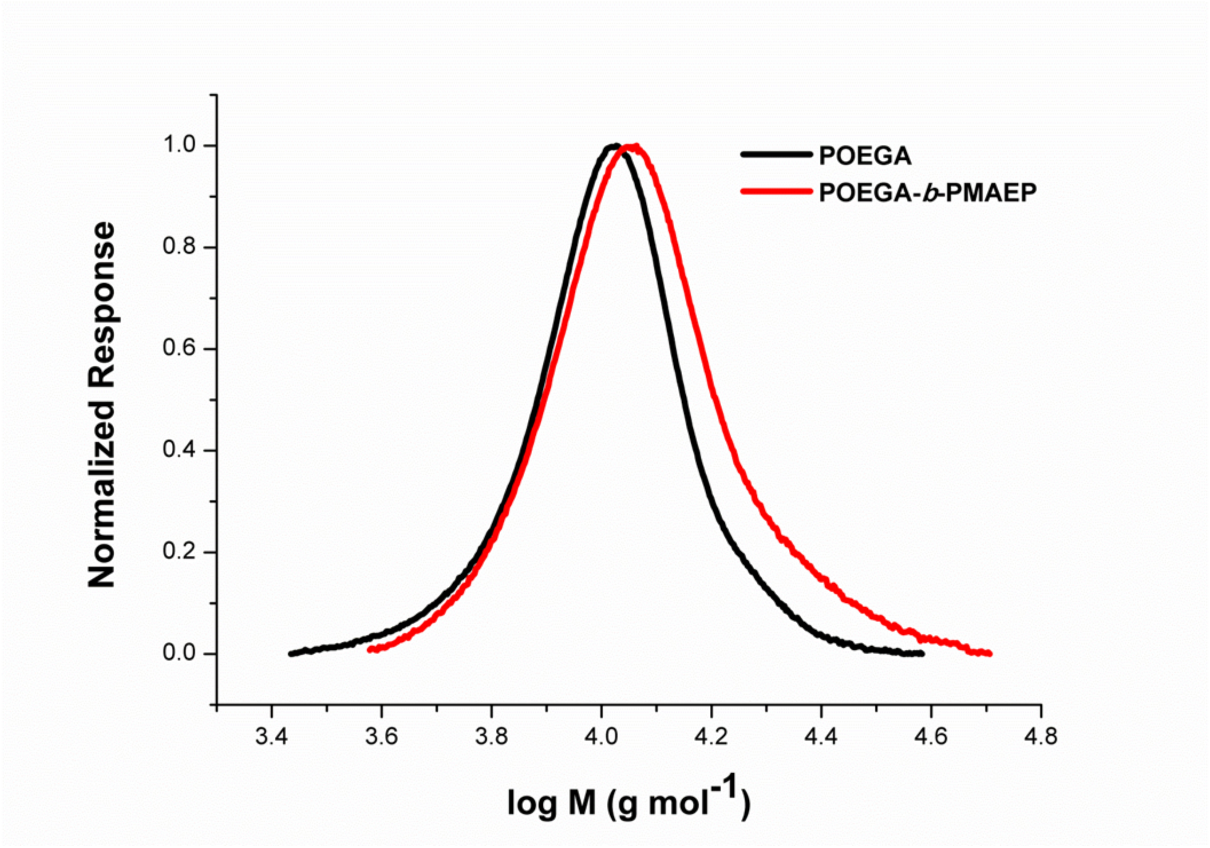

Fig. S3. SEC chromatograms of POEGA and POEGA-*b*-PMAEP

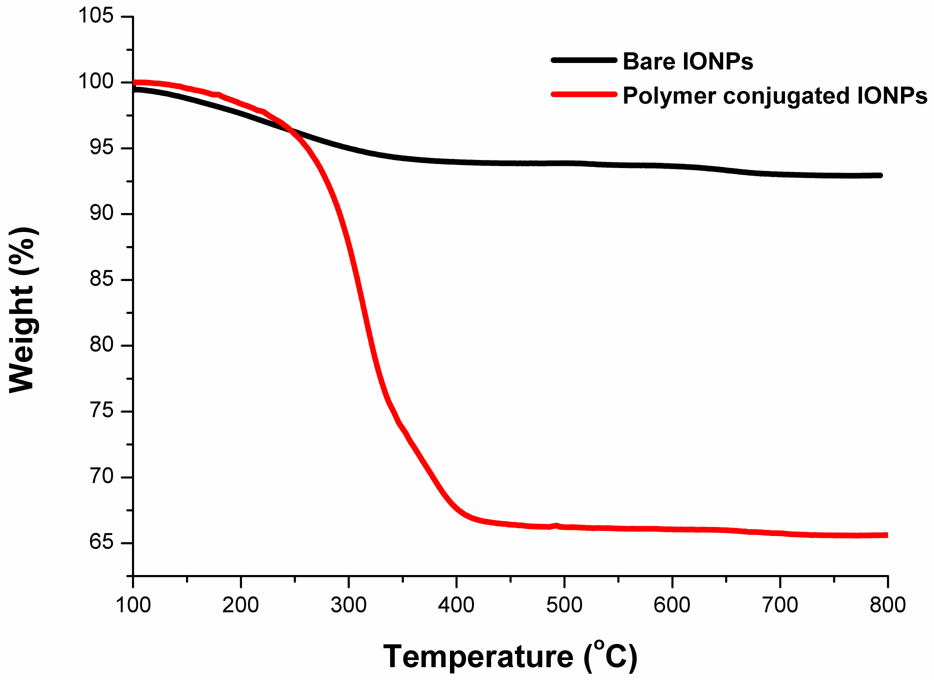

Fig. S4. TGA of bare IONPs and POEGA-*b*-PMAEP@IONPs synthesized using "grafting onto" approach.

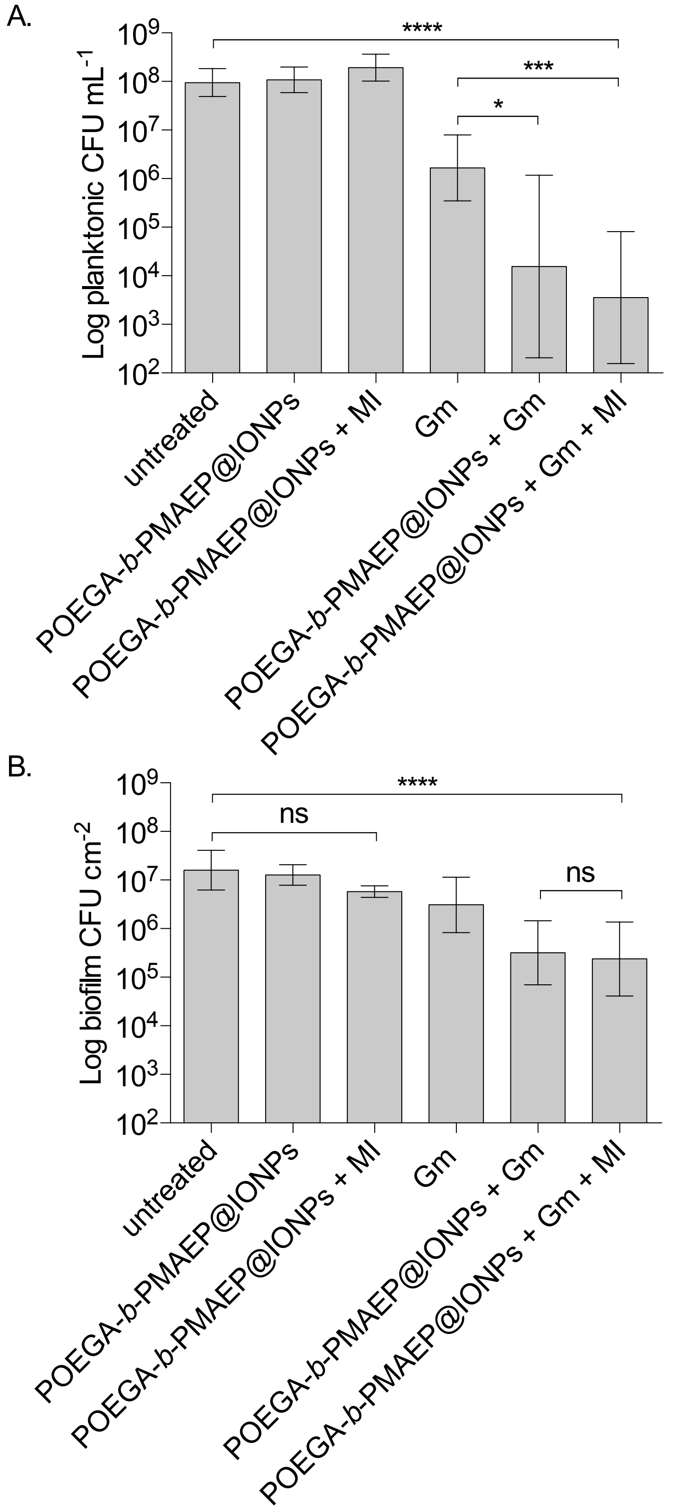

Fig. S5. IONP-induced hyperthermia increases the efficacy of the antibiotic gentamicin against both biofilm and planktonic *P. aeruginosa*. Biofilms were grown as described before for 24 h in test tubes before adding POEGA-*b*-PMAEP@IONPs (1 mg mL^{-1}), gentamicin (Gm, 2 mg mL^{-1}), a combination of both or no treatment to the cultures, and exposing (+ MI) or not the cultures to an alternating magnetic field (6.5 T, 196 kHz) for a further 2 h. After treatment, colony-forming units (CFU) analyses were performed for both the planktonic (A) and biofilm (B) phases. MI, magnetic induction. Error bars represent standard errors ($n \geq 4$). Asterisks indicate statistically significant difference of treatment versus untreated culture (*, $P < 0.1$; ***, $P < 0.001$; ****, $P < 0.0001$; ns, not significant).

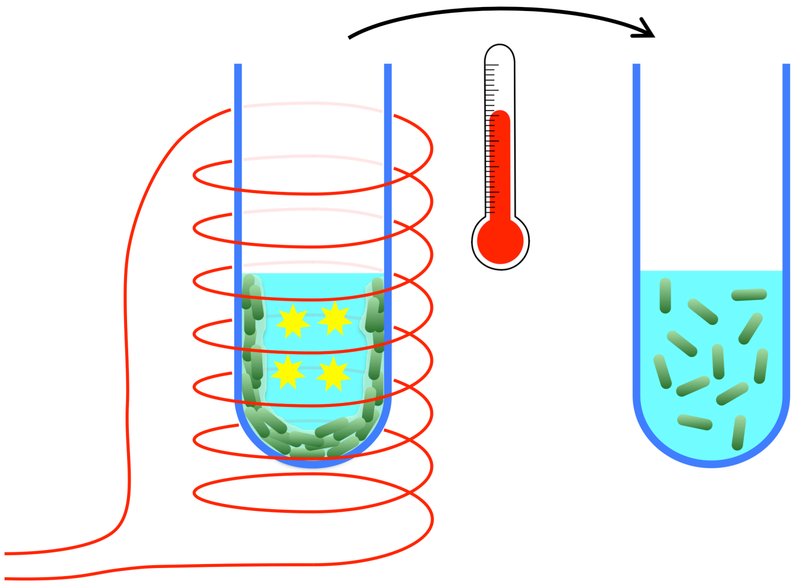

Schematic representation of IONP-mediated hyperthermia inducing biofilm dispersal.
